# Epidemiological characterization of human infection with H5N6 avian influenza

**DOI:** 10.3389/fpubh.2024.1398365

**Published:** 2024-10-02

**Authors:** Fengying Li, Zhou Sun, Mingyong Tao, Kai Song, Zhe Wang, Xiaobin Ren

**Affiliations:** ^1^Department of Clinical Laboratory, Sir Run Run Shaw Hospital, Zhejiang University School of Medicine, Hangzhou, China; ^2^Hangzhou Center for Disease Control and Prevention, Hangzhou, China

**Keywords:** avian influenza, H5N6 subtype, human infection, epidemiological characterization, prognosis

## Abstract

**Background:**

In recent years, there have been frequent reports of human infection with H5N6 avian influenza. However, the fundamental characteristics of the disease remain unclear. This paper conducts a systematic review to explore the epidemiological features of the disease, aiming to provide a foundation for epidemic prevention and control and to serve as a reference for clinical diagnosis.

**Method:**

A systematic search was performed in PubMed, Web of Science, CNKI, Wanfang and gray literature up to November 15, 2023. All articles were about the epidemic features of the H5N6 subtype of avian influenza, written in English or Chinese.

**Results:**

This review encompasses 24 documented outbreaks of human H5N6 avian influenza, exclusively reported in southern China. The age range of cases spanned from under 2 years old to 81 years old. The incubation period ranged from 1 to 13 days, with a mean of 4.3 days. Among the 24 cases, 22 individuals had a documented history of contact with poultry. Of the 23 cases with available prognosis data, 12 resulted in fatalities, yielding a significant fatality rate of 52.2%. A noteworthy observation is that all cases with a history of contact with sick and dead poultry resulted in fatalities, and the difference in fatality rates between this group and others was statistically significant (χ^2^ = 7.441, *p* = 0.014). This study identified a total of 888 close contacts, none of whom demonstrated infection.

**Conclusion:**

This study represents a comprehensive summary of the epidemiological characteristics of human H5N6 avian influenza. Significantly, it sheds light on the incubation period of the disease and underscores a potential elevated risk of mortality among patients with a history of contact with sick and dead poultry.

## 1 Introduction

Influenza A virus (IAV) is the most diverse and epidemiologically significant pathogen associated with severe disease manifestations in humans (1). Wild aquatic birds are the natural reservoirs for IAV, but it can infect a variety of animals, including poultry, aquatic animals (e.g., seals, dolphins, and whales) and terrestrial mammals (e.g., cats, dogs, horses, pigs, mink, tigers, and humans) (2, 3). Taubenberger conducted a complete genome sequence and evolutionary analysis of the 1918 Spanish flu virus, confirming it to be a strain of avian influenza virus adapted entirely to humans, namely the H1N1 avian influenza virus (4). In early 2013, China reported the first cases of human infection caused by a novel H7N9 avian influenza virus (5). Importantly, the low-pathogenic avian influenza virus H7N9 evolved into a highly pathogenic strain. Due to the lack of pre-existing immunity in the majority of the population, infections with these viruses resulted in severe illnesses and fatalities (6).

Coincidentally, in April 2014, the world’s first reported case of human infection with the H5N6 subtype of avian influenza in Sichuan Province, China (7). Later that year, Guangdong Province in China reported a second case of this nature (8). Over the following years, more than 10 provinces in China reported cases of human infections with the H5N6 avian influenza virus. While numerous cases of H5N6 avian influenza infections in humans have been reported, the lack of systematic analyses, primarily in the form of individual case reports, has left essential characteristics of human H5N6 infection unclear. Basic features of this disease, such as its regional and temporal distribution, modes of exposure, incubation period, prognosis for those infected, and potential for human-to-human transmission, remain inadequately understood. Addressing these fundamental epidemiological questions is crucial for clinical diagnosis and the control of outbreaks associated with this disease. This study aims to comprehensively evaluate previous case reports to provide insights into these questions and offer valuable references for clinical diagnosis and epidemic control of H5N6 avian influenza.

## 2 Methods

### 2.1 Search strategy

This review adhered to the Preferred Reporting Items for Systematic Reviews and Meta-Analyses (PRISMA) guidelines (9). All analyses were conducted based on previously published studies, obviating the need for additional ethical approval or patient consent. The literature retrieval process encompassed databases such as WANFANG, CNKI, PubMed, and Web of Science, utilizing search terms including “Human infection,” “H5N6,” and “Highly pathogenic avian influenza” up to November 15, 2023. In addition to database searches, reference lists of the identified articles underwent manual scrutiny for potential inclusion of supplementary studies. Possible gray literature was also searched using Google Scholar to identify additional relevant studies. Additionally, the World Health Organization (WHO) website and regional health department websites were examined to find pertinent articles and reports. Titles and abstracts were screened for relevance by two independent reviewers (Fengying Li and Zhou Sun). In the event of disagreements, a consensus on data extraction was reached through discussion involving a third investigator (Mingyong Tao).

### 2.2 Selection criteria

Inclusion Criteria were as follows: (1) reporting of H5N6 avian influenza outbreaks, including details such as outbreak time, province, the number of cases, age and gender of the case, the number of close contacts and close contacts with the medical observation time, prognostic information, avian exposure history; (2) publications in English or Chinese; (3) positive nucleic acid tests for H5N6 in specimens of the case.

Exclusion criteria included: (1) outbreaks reported due to mixed infections with other pathogens; (2) unavailability of full-text with the inability to collect data from the abstract; (3) insufficient information about the epidemiological characteristics; (4) articles categorized as review articles.

### 2.3 Data extraction

For studies with repeated outbreaks, each instance was counted only once. Duplicated information found across different studies was systematically summarized and integrated. An information extraction table was created in Excel to collect the following details from the qualified literature: first author, reporting province, age, sex, exposure type, exposure time, onset time, prognostic information of the cases, number of close contacts, and medical observation information of the close contacts.

### 2.4 Statistical analysis

The data from the literature were extracted and entered into Microsoft Excel. Calculations for both the percentage and mean values relevant to this study were conducted using Excel software. The correlation analysis concerning age, gender, mode of exposure, and case prognosis was performed employing the statistical software SPSS 13.0 through the application of the chi-square test.

## 3 Results

### 3.1 Identification and selection of studies

**Figure 1:**
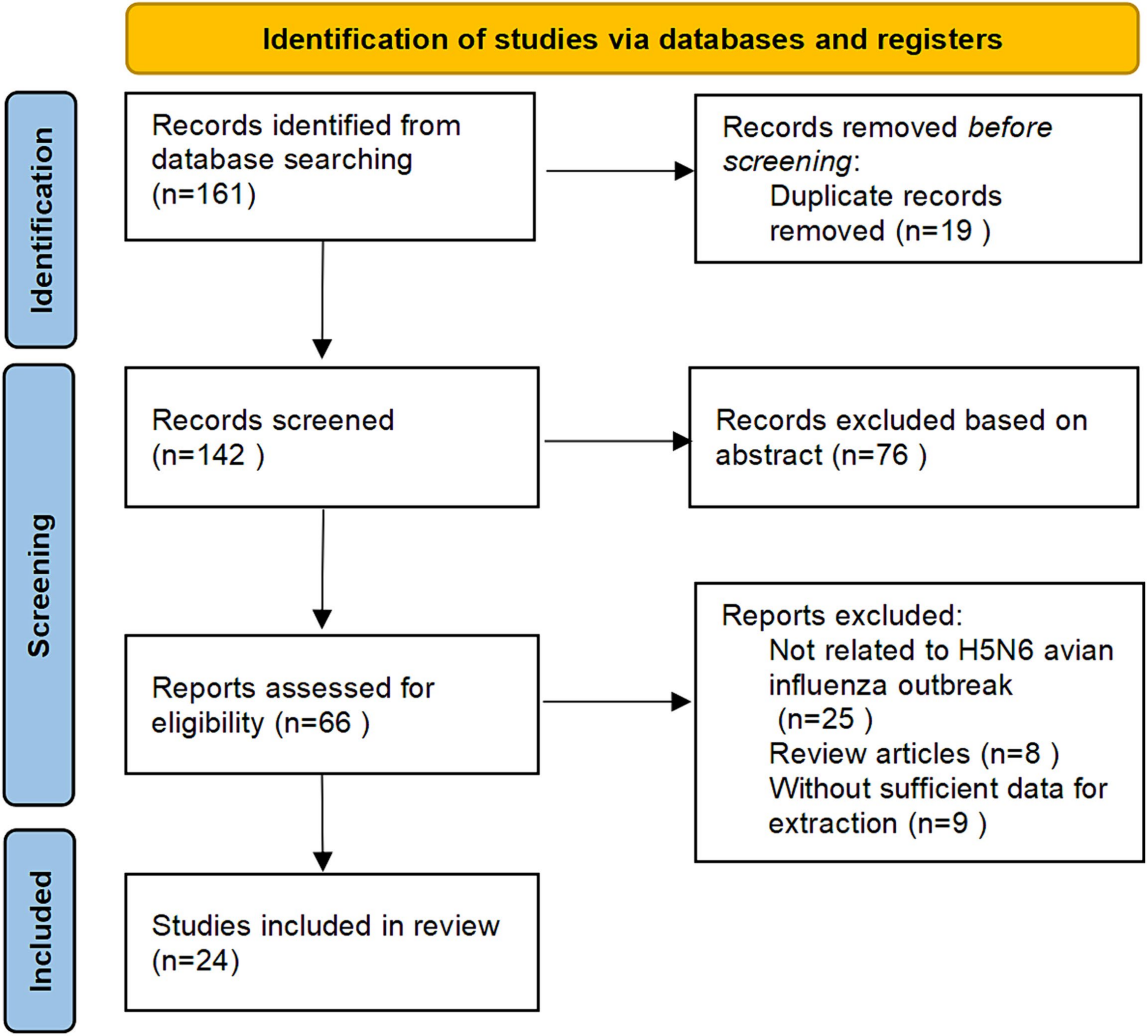
Flow chart for study identification in the systematic literature review of the epidemiological characteristics of human infection with H5N6 avian influenza.

A total of 161 studies were initially identified through a comprehensive primary search. Subsequent to the meticulous removal of 19 duplicate entries and the exclusion of 76 studies deemed irrelevant to the scope of our investigation, the detailed full texts of 66 papers underwent a thorough evaluation for eligibility in this study. From this scrutiny, 42 articles were excluded, comprising 25 studies unrelated to H5N6 avian influenza outbreaks, 8 review articles, and 9 lacking sufficient data for extraction. The study selection process, including the exclusions and the reasons for each, is visually depicted in Figure 1.

The final inclusion comprised 24 published articles, each contributing unique insights into H5N6 avian influenza outbreaks. These outbreaks, totaling 24, were exclusively documented in Chinese territory and occurred during the period from April 2014 to November 2023. A comprehensive overview of the fundamental characteristics of the included literature is presented in Table 1.

**Table 1:** The overview of the fundamental characteristics of the included literature.

First author	Publication year	Province	Case					
			Age	Gender	Exposure types	Exposure time	Onset time	Prognosis
He et al. (28)	2015	Yunnan	44	Male	ETLB	2015/1/24	2015/1/27	Death
Pan et al. (7)	2016	Sichuan	49	Male	ETSDB	None	2014/4/13	Death
Li et al. (29)	2016	Guangdong	58	Male	ETLB	2014/12/2	2014/12/4	Survival
Li et al. (10)	2016	Guangxi	42	Male	ETSDB	None	2015/12/12	Death
Chen et al. (21)	2016	Guangdong	26	Female	ETLB	2015/12/20	2015/12/24	Death
Ye et al. (11)	2016	Guangdong	25	Male	ETLB	None	2016/1/1	Survival
Yao et al. (12)	2016	Hunan	11	Female	ETLB	None	2016/4/5	Death
Jiang et al. (30)	2016	Hubei	35	Male	ECAE	None	2016/4/9	Survival
Wang et al. (31)	2016	Anhui	66	Female	ETSDB	2016/4/11	2016/4/24	Death
Zhang et al. (32)	2018	Anhui	65	Female	ETSDB	2016/4/11	2016/4/15	Death
Meng et al. (33)	2018	Guangxi	30	Female	ETSDB	None	2016/11/14	Death
Liang et al. (34)	2019	Guangxi	33	Male	ETLB	None	2017/11/7	Death
Fan et al. (35)	2019	Guangxi	42	Male	ETLB	None	2018/8/10	Survival
Huang et al. (36)	2020	Beijing	59	Female	ETSB	2019/7/31	2019/8/6	Survival
Liu et al. (24)	2020	Guangdong	22	Male	ETLB	2018/9/24	2018/9/25	Survival
Li et al. (22)	2021	Anhui	1.8	Female	ETLB	2020/12/20	2020/12/22	Survival
Kong et al. (23)	2022	Jiangsu	81	Female	ETLB	2020/11/11	2020/11/16	Death
Tian et al. (37)	2022	Guizhou	3	Female	ETSDB	2020/11/19	2020/11/21	Death
Jiang et al. (13)	2022	Chongqing	52	Male	ETLB	None	2020/12/18	Survival
Chen et al. (14)	2022	Hunan	55	Female	ETLB	None	2021/7/26	None
Mo et al. (38)	2022	Guangxi	3.9	Male	ECAE	None	2021/11/15	Survival
Li et al. (18)	2022	Zhejiang	51	Female	ETSB	2021/12/7	2021/12/15	Survival
Zhang et al. (19)	2022	Jiangsu	6	Female	ETLB	None	2022/1/20	Survival
Jia et al. (39)	2023	Hunan	28	Male	ETLB	2022/3/16	2022/3/17	Death

ETLB: exposure to live birds; ETSDB: exposure to sick and dead birds; ETSB: exposure to slaughtered birds; ECAE: no clear avian exposure. None: information was not mentioned; NO.: the number.

### 3.2 Epidemiological characteristics

#### 3.2.1 Distribution of the age and gender

In the 24 documented cases, the age range spanned from less than 2 years to 81 years, with a mean age of 37 years. The distribution across age groups revealed that the majority of cases, 16 in total, fell within the young adult category (20–60 years old), constituting 66.7% of the cases. Five cases were under 20 years old, representing 20.8%, while an additional 5 cases were over 60 years old, accounting for 12.5% of the total. Gender-wise, the cases were evenly distributed, with 12 cases each reported for both men and women.

#### 3.2.2 Regional distribution

In the preceding period, H5N6 avian influenza outbreaks were documented across 14 provinces or municipalities (including Beijing, and Chongqing) in China. Notably, Guangdong and Guangxi provinces exhibited the highest incidence, each contributing 4 out of 24 cases, representing 16.7% of the total. Following closely were Anhui with 3 cases (12.5%) and Hunan and Jiangsu with 2 cases each (8.3% each). The remaining provinces or municipalities, including Beijing, Guizhou, Henan, Hubei, Jiangxi, Sichuan, Yunnan, Zhejiang, and Chongqing, reported a single case each, constituting 4.2% each.

Our investigation revealed a predominant concentration of outbreaks in southern China (south of the Qinling Mountains and the Huai River). Furthermore, all documented outbreaks occurred within the southeastern China (from Heihe City in Heilongjiang province to Tengchong City in Yunnan province, effectively dividing China into two distinct halves—southeast and northwest).

**Figure 2:**
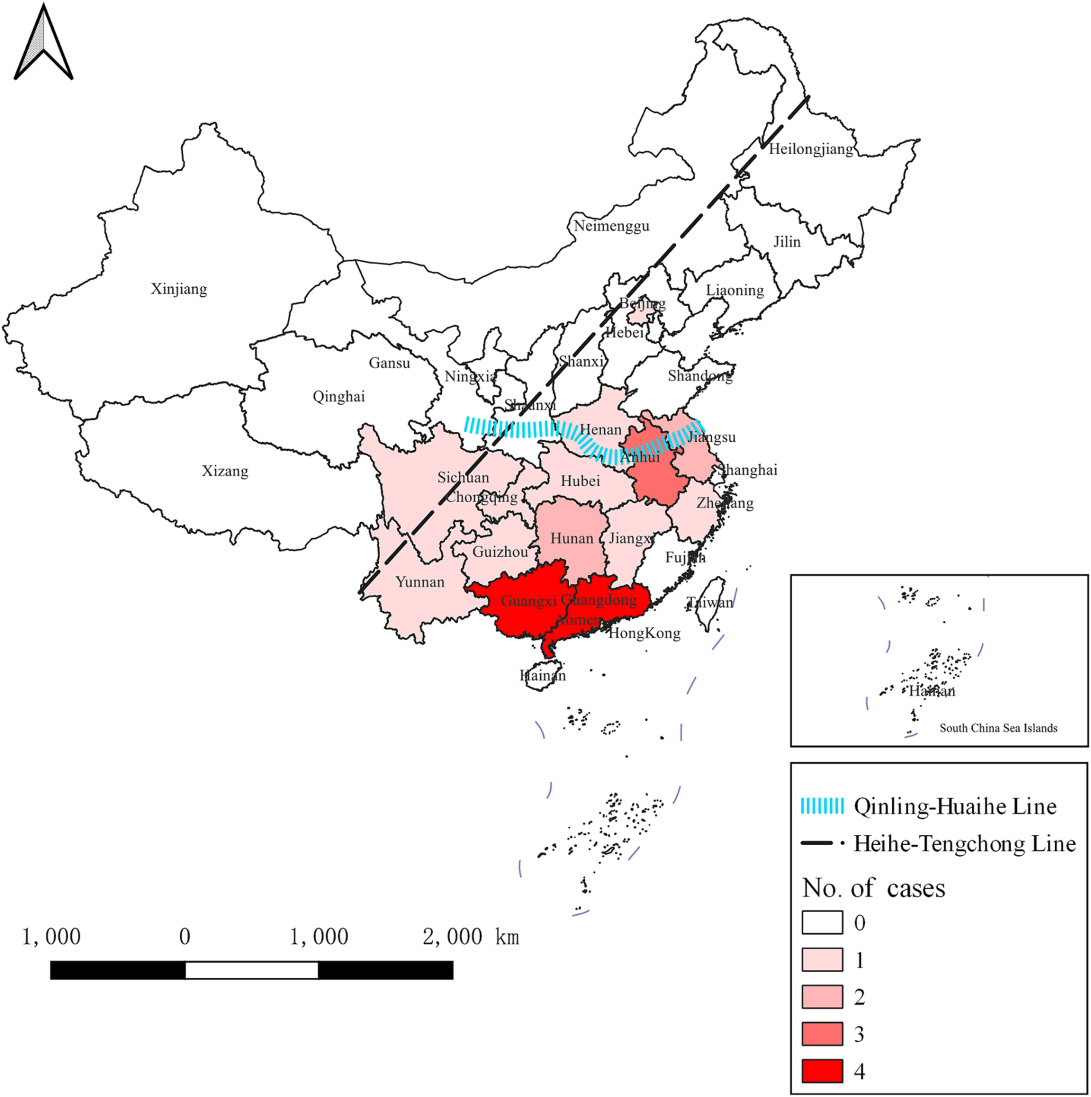
Regional distribution of H5N6 avian influenza outbreaks in China from April 2014 to November 2023. The dotted black line is the Hu line, which divides China into two parts, namely southeast and northwest. The blue line represents the Qinling Mountains and the Huai River, which divides China into the southern and northern parts. H5N6 avian influenza outbreaks were documented across 14 provinces or municipalities. The darker the color, the greater the number of outbreaks in the province.

The regional distribution of H5N6 avian influenza outbreaks is shown in Figure 2.

#### 3.2.3 Temporal distribution

All 24 outbreak reports meticulously documented the time of patient onset. The earliest outbreak was recorded in April 2014, while the most recent occurred in March 2022, creating a comprehensive temporal span for our analysis. The zenith of outbreaks transpired in 2016, constituting 25.0% of the total cases, followed by 2020 with 4 cases (16.7%), 2015 with 3 cases (12.5%), and 2021 also with 3 cases (12.5%). The years 2014 and 2018 each accounted for 2 cases (8.3% each), and 2022 similarly contributed 2 cases (8.3%). Both 2017 and 2019 reported a single case each, representing 4.2% each of the total cases.

**Figure 3:**
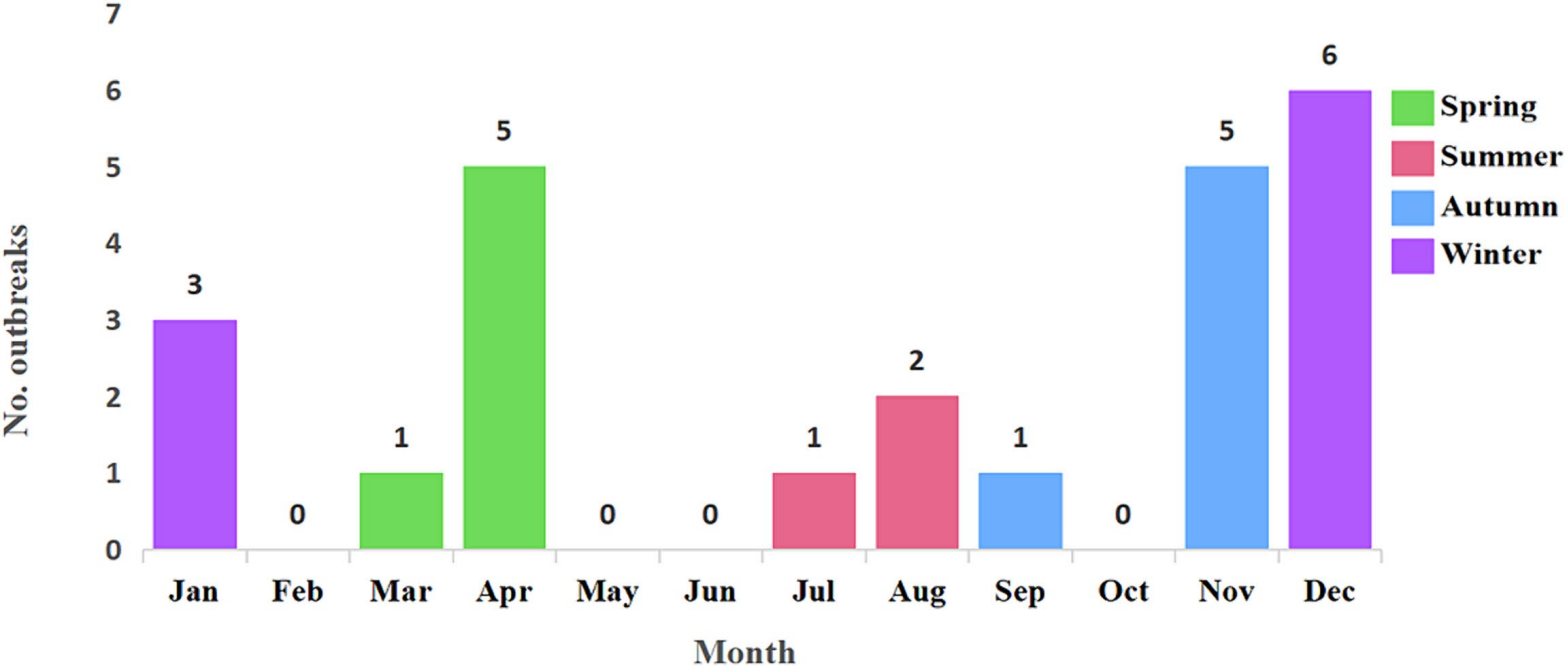
Temporal distribution of 24 H5N6 avian influenza outbreaks from April 2014 to November 2023. The 24 H5N6 avian influenza outbreaks occurred in every season throughout the studied period. There were 9 outbreaks in winter, 6 outbreaks each in spring and autumn, and 3 outbreaks in summer. This pattern generally aligns with the seasonal distribution characteristics of other respiratory infections, showing a peak in winter, followed by spring and autumn, with the fewest outbreaks occurring in summer.

H5N6 avian influenza outbreaks occurred in every season throughout the studied period. Specifically, there were 9 outbreaks in winter (December to February), constituting 37.5% of the total cases. Spring (March to May) and autumn (September to November) each reported 6 outbreaks, accounting for 25.0% of the total cases, respectively. In summer (June to August), there were 3 outbreaks, contributing to 12.5% of the total cases. Basically, it is consistent with the seasonal distribution characteristics of other respiratory transmission, high in winter, followed in spring and autumn, and least in summer. A detailed monthly breakdown of the number of cases is provided in Figure 3.

#### 3.2.4 Exposure and incubation period

Based on the actual exposure history of the 24 cases, we categorized the avian exposure into four distinct types: exposure to infected sick and dead birds, exposure to infected live birds, exposure to infected slaughtered birds, and no clear avian exposure history. Impressively, 22 out of the 24 cases, representing 91.7%, had a clearly documented history of avian exposure. Only 2 cases lacked a clear avian exposure history. Among the cases with clear exposure history, the majority had been exposed to live poultry, accounting for 58.3%. A significant portion had a history of exposure to sick and dead birds, representing 25.0%, while the least number of cases were exposed to slaughtered poultry, accounting for 8.3%.

**Figure 4:**
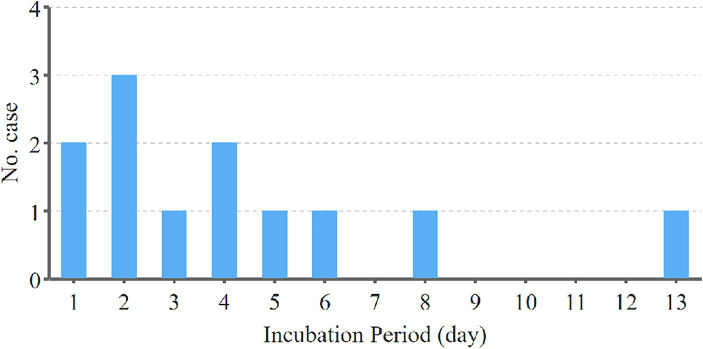
The number of cases with different incubation period in the cohort of 12 H5N6 cases with a clear and singular exposure history. The incubation period of H5N6 avian influenza was calculated from the time of patient exposure to the earliest manifestation of clinical symptoms, measured in days. Among these 12 cases, the shortest incubation period observed was 1 day, while the longest recorded was 13 days. The majority of cases exhibited a relatively short incubation period, with 10 out of the 12 cases developing symptoms within 1 week. No cases displayed an incubation period exceeding 2 weeks.

In the process of calculating the latency of H5N6 avian influenza, we refined our analysis by excluding cases with unclear exposure history and continuous exposure. This yielded a dataset comprising only cases with a single, well-documented exposure history, resulting in a cohort of 12 cases with a clear and singular exposure history. The incubation period of H5N6 avian influenza was then calculated from the time of patient exposure to the earliest manifestation of clinical symptoms, measured in days. Notably, among these 12 cases, the shortest incubation period observed was 1 day, while the longest recorded was 13 days. The mean incubation period across these cases was determined to be 4.3 days. Remarkably, the majority of cases exhibited a relatively short incubation period, with 10 out of the 12 cases (83.3%) developing symptoms within 1 week. Importantly, no cases in the dataset displayed an incubation period exceeding 2 weeks. The number of cases with different incubation period is shown in Figure 4.

#### 3.2.5 Prognosis of the cases

Among the 24 documented outbreaks, 23 provided clear prognostic information, while details on one case were not mentioned. Of the 23 cases with available prognostic data, 11 individuals survived, and 12 died. Calculating based on this information, the survival rate for H5N6 cases was determined to be 47.8% (11 out of 23), while the corresponding fatality rate stood at 52.2% (12 out of 23).

In our effort to discern prognostic factors associated with H5N6 influenza cases, we conducted a comprehensive analysis examining the interplay between age, sex, mode of exposure, and case prognosis. Notably, all cases over 60 years old resulted in fatalities, implying a seemingly elevated case fatality rate in this age group. While this aligns with expectations, considering the likelihood of concurrent underlying diseases in older individuals, our statistical analysis did not reveal a significant difference (χ^2^ *=* 3.162, *p =* 0.217), potentially due to the limited sample size.Among the female cases, 7 out of 11 had succumbed to the infection, yielding a case fatality rate of 63.6%, surpassing that of male cases (41.7%, 5 out of 12). However, no statistically significant difference was observed between the two genders (χ^2^ *=* 1.110, *p =* 0.414). A noteworthy finding emerged concerning cases with a history of exposure to sick and dead birds, where the case fatality rate was 100% (6 out of 6). This was markedly higher than the fatality rate in other exposure groups (35.3%, 6 out of 17), and the difference was statistically significant (χ^2^ *=* 7.441, *p =* 0.014). This discrepancy may be attributed to the potentially higher viral load encountered by cases with exposure to sick and dead birds. The detailed statistical results are presented in Table 2.

**Table 2:** The analysis between age, gender, mode of exposure, and case prognosis.

	Survival	Death	χ^2^	*P*-value
Age				
<60	11	9	3.162	0.217
≥60	0	3		
Gender				
Male	7	5	1.110	0.414
Female	4	7		
Exposure				
Sick and dead birds	0	6	7.441	**0.014**
Others	11	6		

Others: exposure to live birds and exposure to slaughtered birds and no clear avian exposure.

Bold value: *p* ≤ 0.05, with significant difference.

#### 3.2.6 Close contacts for medical observation

In the context of 24 outbreaks of H5N6 avian influenza, it was explicitly indicated that there were 20 cases with a specified number of close contacts. Among these, 4 cases underwent a specified 14-day medical observation period for close contacts, 4 cases had a 10-day observation period, and 9 cases had a 7-day observation period. The study encompassed a total of 888 close contacts, yet no instances of infection were identified among them.

## 4 Discussion

In this study, we determined for the first time that the shortest incubation period for H5N6 avian influenza in humans is 1 day, the longest is 13 days, and the average is 4.3 days. When calculating the incubation period of the disease, we typically need to know the specific single exposure time of the patient, so we excluded cases with continuous exposure (7, 10–14). In the end, only 12 cases met our criteria for calculating the incubation period. Understanding the incubation period of H5N6 avian influenza is crucial for clinical diagnosis and treatment. After being infected with the H5N6 avian influenza virus, cases often present with symptoms such as fever, cough, and unexplained pneumonia upon hospital admission. This underscores the importance for clinicians to consider the patient’s poultry contact history during the incubation period to enable earlier diagnosis and timely administration of oseltamivir, thereby reducing the mortality rate (15). Additionally, co-exposed persons and close contacts of cases usually require medical observation, and the incubation period is the premise for determining the duration of medical observation and is also an important part of epidemic control. Based on the calculated incubation period, and considering the severity of the prognosis for this disease, we believe that a two-week medical observation period for this disease is reasonable. Additionally, Zhou et al. using data from cases-patient groups in China from 2013 to 2017, estimated the incubation period of H7N9 avian influenza virus infection, with a median incubation period of 4 days (16). This indicates that the incubation period of H5N6 avian influenza is similar to that of H7N9 avian influenza.

We calculated the case fatality rate of H5N6 avian influenza to be 52.2%, which is relatively high compared to the H7N9 subtype of avian influenza and is generally consistent with the H5N1 subtype of avian influenza (17). It is worth mentioning that we discovered for the first time that the case fatality rate of cases with exposure history to sick and dead poultry and dead birds is 100%, which is significantly higher than that of other exposure groups. This may be related to the fact that sick and dead poultry have more virus loads. It also reminds us that avian flu patients exposed to sick and dead birds have a higher risk of death.

In current systematic review, we categorized the prognosis of cases into two groups: death and survival, rather than death and recovery. Mainly because some cases, although discharged after clinical treatment, left severe sequelae. As the Zhejiang cases occurred in Hangzhou, our team learned through follow-up that the cases experienced the invasion of the H5N6 avian influenza virus, which led to severe and irreversible fibrosis in the lungs, resulting in a very poor physical condition. The cases often experienced pulmonary bleeding and were unable to engage in physical labor in daily life (18). One case in Jiangsu Province exhibited early symptoms of encephalitis, such as seizures, epilepsy, and coma. After being diagnosed with H5N6 virus infection, the patient underwent treatment with oseltamivir and received methylprednisolone pulse therapy. However, during the 2–4 month follow-up after discharge, obvious characteristics of posterior cerebral atrophy was observed in the patient (19). This indicates that in addition to having a very high fatality rate after infection with H5N6, some cases also have severe sequelae. In general, the H5N1, H5N6, H7N9, and H10N8 subtypes of avian influenza have a relatively high risk of death compared to other subtypes (20).

In this research, we classified exposure into four categories: exposure to infected and dead birds, exposure to infected live poultry, exposure to infected slaughtered poultry, and no clear history of poultry exposure. The essence of this classification is based on the patient’s exposure object, rather than the traditional classification based on exposure pathways. The main reason is that for some cases, the exposure object is clear, while the exposure pathway may be mixed, making it impossible to determine which pathway caused the infection. For example, in the articles by Chen, Li, and Kong, it was only investigated that the cases had slaughtered live poultry a few days before, making it impossible to determine whether the infection occurred through respiratory exposure to the H5N6 virus during the slaughtering process or through contact exposure (21–23). Similarly, Liu’s study suggested that the cases were infected with avian influenza virus through aerosol transmission (24). We believe that using the classification method of exposure objects avoids subjective errors introduced by classifying exposure pathways and is more practically meaningful for epidemic prevention and control.

Our research includes 888 close contacts, who have undergone a medical observation of at least 7 days. However, no instances of infection were found among these close contacts. Due to the limited number of cases in our study, we cannot yet rule out the possibility of human-to-human transmission of the disease. Nevertheless, our data suggests that the human-to-human transmission capability of H5N6 avian influenza is limited. In contrast to the H5N6 avian influenza outbreak, there have been more reports of H7N9 outbreaks (25), some of which include reports of illness among close contacts and instances of familial clustering (26, 27).

While our research results can provide a basis for the prevention and control of H5N6 avian influenza, as well as provide references for clinical diagnosis and treatment, our systematic review also has certain limitations. Firstly, the sample size of our study is not large enough. Secondly, our article lacks some clinical treatment information, making it challenging to provide sufficient support for clinical treatment. Additionally, case ascertainment may be influenced by the healthcare infrastructure, diagnostic criteria, and reporting practices of the region. In this review, the reliance on data from China means the findings may not be fully generalizable to other regions with different healthcare systems and practices. Moreover, the limitation to published cases may introduce the risk of publication bias. Thus, more reliable conclusions will require additional data in the future.

## Data Availability

The original contributions presented in the study are included in the article/supplementary material, further inquiries can be directed to the corresponding author.

## References

[1] Shindo N Briand S. Influenza at the beginning of the 21st century. Bull World Health Organ. (2012) 90:247–247A. doi: 10.2471/BLT.12.104653, PMID: 22511816 PMC3324879

[2] Cinatl J Michaelis M Doerr HW. The threat of avian influenza A (H5N1). Part I: epidemiologic concerns and virulence determinants. Med Microbiol Immunol. (2007) 196:181–90. doi: 10.1007/s00430-007-0042-5, PMID: 17492465

[3] Horimoto T Kawaoka Y. Influenza: lessons from past pandemics, warnings from current incidents. Nat Rev Microbiol. (2005) 3:591–600. doi: 10.1038/nrmicro1208, PMID: 16064053

[4] Taubenberger JK Reid AH Lourens RM Wang R Jin G Fanning TG. Characterization of the 1918 influenza virus polymerase genes. Nature. (2005) 437:889–93. doi: 10.1038/nature04230, PMID: 16208372

[5] Wu A Su C Wang D Peng Y Liu M Hua S . Sequential reassortments underlie diverse influenza H7N9 genotypes in China. Cell Host Microbe. (2013) 14:446–52. doi: 10.1016/j.chom.2013.09.001, PMID: 24055604

[6] Li C Chen H. H7N9 Influenza Virus in China. Cold Spring Harb Perspect Med. (2021) 11:a038349. doi: 10.1101/cshperspect.a038349, PMID: 32205415 PMC8327827

[7] Pan M Gao R Lv Q Huang S Zhou Z Yang L . Human infection with a novel, highly pathogenic avian influenza A (H5N6) virus: virological and clinical findings. J Infect. (2016) 72:52–9. doi: 10.1016/j.jinf.2015.06.009, PMID: 26143617

[8] Yang ZF Mok CK Peiris JS Zhong NS. Human infection with a novel avian influenza A (H5N6) virus. N Engl J Med. (2015) 373:487–9. doi: 10.1056/NEJMc1502983 26222578

[9] Page MJ McKenzie JE Bossuyt PM Boutron I Hoffmann TC Mulrow CD . The PRISMA 2020 statement: an updated guideline for reporting systematic reviews. Syst Rev. (2021) 10:89. doi: 10.1186/s13643-021-01626-4, PMID: 33781348 PMC8008539

[10] Li R Liao Y Xie Y Li JH Huang RF Li HP . Epidemiological analysis of the first avian A (H5N6)influenza case in Jiang Xi Province, Anhui. J Prev Med. (2016) 22:301–55.

[11] Bi Y Ji Y Liu FR Li G Liu F. Investigation and analysis of one case of human infected with H5N6 avian influenza virus in Longgang District of Shenzhen City. Trop Med. (2016) 16:728–32. doi: 10.13604/j.cnki.46-1064/r.2016.07.27

[12] Yao ZY Lu XL Luo RP. Severe H5N6 avian influenza pneumonia: first child case in China. Chin J Pract Pediatr. (2016) 31:524–7. doi: 10.7504/ek2016070612

[13] Jiang H Xie JW Xiong HL Fan AH Yang L Luo MY. Epidemiological investigation on the first human case of H5N6 avian influenza in Chongqing. Parasitoses Infect Dis. (2022) 20:56–60.

[14] Chen BT Zheng W Chen BL Xiao J Liu X. Epidemiological investigation and analysis of the first human infection case of H5N6 avian influenza in Chenzhou City, Hunan Province in 2021. Med Health. (2022) 4:220–2.

[15] Kosasih H Bratasena A Pangesti K Laras K Samaan G. Managing seasonal influenza: oseltamivir treatment policy in Indonesia? Acta Med Indones. (2014) 46:58–65. PMID: 24760811

[16] Zhou L Li Q Uyeki TM. Estimated incubation period and serial interval for human-to-human influenza A (H7N9) virus transmission. Emerg Infect Dis. (2019) 25:1982–3. doi: 10.3201/eid2510.190117, PMID: 31264568 PMC6759274

[17] Liu S Sha J Yu Z Hu Y Chan TC Wang X . Avian influenza virus in pregnancy. Rev Med Virol. (2016) 26:268–84. doi: 10.1002/rmv.1884 27187752

[18] Li J Fang Y Qiu X Yu X Cheng S Li N . Human infection with avian-origin H5N6 influenza A virus after exposure to slaughtered poultry. Emerg Microbes Infect. (2022) 11:807–10. doi: 10.1080/22221751.2022.2048971, PMID: 35234570 PMC8920390

[19] Zhang L Liu K Su Q Chen X Wang X Li Q . Clinical features of the first critical case of acute encephalitis caused by the avian influenza A (H5N6) virus. Emerg Microbes Infect. (2022) 11:2437–46. doi: 10.1080/22221751.2022.2122584, PMID: 36093829 PMC9621215

[20] Wang W Chen X Wang Y Lai S Yang J Cowling BJ . Serological evidence of human infection with avian influenza A (H7N9) virus: a systematic review and Meta-analysis. J Infect Dis. (2022) 226:70–82. doi: 10.1093/infdis/jiaa679, PMID: 33119755 PMC9373149

[21] Chen B Zhong ZP Li Y Rao D Liu H Yu H . First cases report of human avian influenza A (H5N6) virus infection in Shenzhen city. J Trop Med. (2016) 16:1581–4. doi: 10.3969/j.issn.1672-3619.2016.12.032

[22] Li JW Ding ZT Sun L Bo G He J Li HB . Epidemiological investigation on a case of child infection with avian influenza A (H5N6) virus. Anhui J Prevent Med. (2021) 27:208–11. doi: 10.19837/j.cnki.ahyf.2021.03.010

[23] Kong WR Yu X Shi YQ Song MY. Epidemiological investigation and Management of the First Human Infection Case of H5N6 avian influenza in Liyang City. Jiangsu J Prevent Med. (2022) 33:82–3. doi: 10.13668/j.issn.1006-9070.2022.01.028

[24] Liu YH Lu JY Liu W, H Ma Y Cao L Li KB Li TG Zhang ZB Yang ZC Epidemiological characteristics of a case infected with avian influenza A (H5N6) virus associated with exposure to aerosol. Chin J Epidemiol: (2020), 41:358–362. doi: 10.3760/cma.j.issn.0254-6450.2020.03.015, PMID: 32294835

[25] Liu WJ Xiao H Dai L Liu D Chen J Qi X . Avian influenza A (H7N9) virus: from low pathogenic to highly pathogenic. Front Med. (2021) 15:507–27. doi: 10.1007/s11684-020-0814-5, PMID: 33860875 PMC8190734

[26] Qi X Qian YH Bao CJ Guo XL Cui LB Tang FY . Probable person to person transmission of novel avian influenza A (H7N9) virus in eastern China, 2013: epidemiological investigation. BMJ. (2013) 347:f4752. doi: 10.1136/bmj.f4752, PMID: 23920350 PMC3805478

[27] Jie Z Xie J He Z Song Y Hu Y Li F . Family outbreak of severe pneumonia induced by H7N9 infection. Am J Respir Crit Care Med. (2013) 188:114–5. doi: 10.1164/rccm.201304-0797LE, PMID: 23815728

[28] He JB Duan J Zheng Y. First human case of avian influenza A (H5N6) in Yunnan province, China. SAGE Open Med Case Rep. (2015) 3:2050313X1559648. doi: 10.1177/2050313X15596484, PMID: 27489694 PMC4857328

[29] Li K Liu H Yang Z Li T di B Chen Z . Clinical and epidemiological characteristics of a patient infected with H5N6 avian influenza A virus. J Clin Virol. (2016) 82:20–6. doi: 10.1016/j.jcv.2016.06.004 27395033

[30] Jiang WJ Zhuang FJ Hou M Wang XY. Investigation and analysis on the first clinical case of human infected with avian influenza (H5N6) in Hubei Province. Prev Med. (2016) 43:4417.

[31] Wang H Xia ZC Chen XL Zhang J. Epidemiological investigation on the first case of human avian influenza A (H5N6) virus infection in Anhui Province. Prev Med. (2016) 22:149–93.

[32] Zhang J Hou S Wu JB Shi YL He J Wu GJ . Investigation and analysis on the first case of human infected with avian influenza (H5N6) in Anhui Province. Chin J Zoonoses. (2018):188–91. doi: 10.3969/j.issn.1002-2694.2018.00.020

[33] Meng AS. Investigation and management of an H5N6 avian influenza human infection case in Rong'an county, Guangxi. Appl Prevent Med. (2018) 24:323–5.

[34] Liang ZL He JZ Tan WF Ding X. Clinical characteristics and epidemiological analysis of the first human death from H5N6 avian influenza in Guigang, Guangxi. China Trop Med. (2019) 19:591–4. doi: 10.13604/j.cnki.46-1064/r.2019.06.22

[35] Fan PC Mao WC Qin JK Weng FQ Huang XF Wei MY . Epidemiological investigation and analysis of the first human infection case of H5N6 avian influenza in Laibin City. Prev Med. (2019) 25:223–7. doi: 10.3969/j.issn.1673-758X.2019.03.016

[36] Huang LY Zhang FL Ge S. Epidemiological investigation of the first confirmed human case of avian influenza A (H5N6) virus infection in Beijing. Int J Virol. (2020) 27:381–4. doi: 10.3760/cma.j.issn.1673-4092.2020.05.007

[37] Tian JZ. Epidemiological investigation, management, and analysis of the first human infection case of H5N6 avian influenza in Huangping. Med Health. (2022) 4:250–3.

[38] Mo SY Tan SM Fang YP Tan HL. A case report of critical H5N6 avian influenza encephalitis in a child successfully treated in Guangxi. China Trop Med. (2022) 22:786–90. doi: 10.13604/j.cnki.46-1064/r.2022.08.19

[39] Jia W Hou RJ Wang LZ Wang LB Jiao XC Zhao JJ . Pidemiological investigation on the first case of human infection with H5N6 avian influenza in Henan. Modern Dis Prevent Control. (2023) 34:371–3.

